# A model-based investigation into urban-rural disparities in tuberculosis treatment outcomes under the Revised National Tuberculosis Control Programme in India

**DOI:** 10.1371/journal.pone.0228712

**Published:** 2020-02-14

**Authors:** Himanshu Singh, Varun Ramamohan

**Affiliations:** Department of Mechanical Engineering, Indian Institute of Technology Delhi, New Delhi, India; London School of Hygiene and Tropical Medicine, UNITED KINGDOM

## Abstract

In this study, we investigate differences in tuberculosis (TB) treatment outcomes between urban and rural India and estimate their impact on epidemiological outcomes such as TB incidence, prevalence and mortality using a mathematical model of TB transmission dynamics. Publicly available district-level treatment outcomes data for new and previously treated TB cases was analyzed in conjunction with census data providing the proportion of urban population in each district to determine the effect of urbanity/rurality on treatment outcomes. Districts were grouped in clusters based on the proportion of urban population in each district, wherein the clusters were identified by applying machine learning methods. Regression analyses revealed that average treatment success rates among both new and previously treated cases decline with increase in the proportion of urban population in a district cluster, with substantially sharper declines in treatment success rates with degree of urbanity observed for previously treated cases. The impact of differences in treatment outcomes on epidemiological outcomes was estimated using a dynamic transmission model developed for this purpose. For example, the cluster with highest treatment success rates is projected to have an average of 3.2% fewer deaths per 100,000 population in comparison with the national average across 2019–24, and the cluster with the lowest treatment success rates has an average of 4.5% more deaths per 100,000 in comparison with the national average. We anticipate that these disparities in TB treatment outcomes and epidemiology between urban and rural India may motivate investigations into the associated causes and their redressal.

## Introduction

India has the highest number of tuberculosis (TB) cases in the world, accounting for over 20% of global incident cases, and an estimated mortality of 480,000 people in 2015 [1,2]. In 1992 the Government of India developed and implemented the Revised National TB Control Programme (RNTCP) to revitalize the erstwhile National TB Programme (NTP). The RNTCP was developed to address the shortcomings of the NTP, which included managerial weaknesses, undue emphasis on X-rays for diagnosis, frequent drug shortages, and underutilization of laboratory services [3]. A major component of the RNTCP involved adopting the internationally recommended TB treatment strategy referred to as Direct Observed Treatment, Short Course (DOTS). The RNTCP has had a significant impact, with TB prevalence reducing from 338 per 100,000 in 1997 to 249 per 100,000 populations in 2009, and mortality reducing from 55 per 100,000 in 2000 to 36 per 100,000 in 2015 [1].

RNTCP was extended to most districts in India by 2006 [3]. However, there remain substantial differences in access to health and socioeconomic conditions in general between urban and rural areas in India. For instance, 72.2% of India’s population resides in rural areas, whereas only 40.8% of health workers practice in rural areas [4]. Rural areas also lag economically, with the per capita income in urban areas being around 2.5 times that in rural areas [5]. Given these differences, we investigate whether there are differences in TB treatment outcomes as well between urban and rural areas. Our study was further motivated by the findings of a study [6] that suggests that RNTCP outcomes in remote, highly forested districts with significant populations of indigenous tribes may not be optimal, with less than half of these districts achieving the target cure rate of 85%. Estimating the extent of these differences is a step towards understanding the impact of improving the treatment programmes in such areas.

We utilize most recently published district-level data for RNTCP treatment outcomes in conjunction with census data for the proportion of a district’s population residing in urban areas for our analysis (referred to hereafter as degree of urbanity). We conduct regression analyses between treatment outcomes and degree of urbanity by using this data in a standalone manner, and also by applying a machine learning method to create clusters of districts based on similarities in their degrees of urbanity. We then estimate how treatment outcomes vary within and across these district clusters. Further, we utilize a model of TB transmission dynamics, which we developed and calibrated to published incidence and prevalence trends, to develop projections and conduct simulation analyses around incidence, prevalence, and mortality trends for each cluster.

We develop this TB transmission dynamics model by substantially modifying Mandal and colleagues’ model [7], to incorporate across-cluster differences in outcomes between previously treated cases and new cases. Multiple TB transmission dynamics models have been developed [7–12], including a few for the Indian context [7,10,11]. Suen and colleagues [10] developed a fairly comprehensive model of TB transmission dynamics, but do not model TB dynamics among previously treated cases to the extent that we require. Further, the degree of detail captured in other aspects of their model (e.g., age and gender stratification) was not required for our purposes, and hence we chose to modify another more recently published model by Mandal and colleagues [7].

In the following sections, we analyze how treatment outcomes depend on the degree of urbanity of a district, and also estimate the cluster-specific probability of being treated for TB in a district with a treatment success rate below the national average. We then construct cluster-wise incidence, prevalence, and mortality projections up to five years in the future, which in turn map to estimates of epidemiological trends for specific districts. We also utilize the model to understand how within-cluster variation in treatment outcomes propagates to variation in epidemiological outcomes and estimate the probabilities of clusters having epidemiological outcomes worse than that of the national average. We anticipate that our analysis can assist stakeholders in identifying districts with RNTCP programmes that require particular attention and help motivate improvement of treatment outcomes in these districts.

## Methods

### Treatment outcomes data analysis

We begin our analysis by studying annual reports of tuberculosis control in India published by the Central TB Division. These reports provide a comprehensive assessment of TB control activities in India under RNTCP. Specifically, they provide quantitative indicators of state and district wise RNTCP performance during the preceding calendar year. We chose the TB India 2014 [13] report for our analysis as we required treatment outcomes at district level resolution under RNTCP and the 2014 report is the most recent report which provides this data (later reports do not). Therefore, we extract treatment success rates for new smear-positive cases and for previously treated smear-positive cases for each district from the report. Note that these were the only district-level treatment success rates reported; for example, treatment-success rates for smear-negative cases were not reported. Further, we were unable to discern, from the information in the report, whether these cases include those with drug-resistant TB; therefore, for the first part of the analysis (without using the dynamic transmission model) we analyze the data as extracted from the report and make no assumptions regarding the patient population associated with the data. However, when we use the treatment success rates from the TB India report in the dynamic transmission model, we make the conservative assumption that the treatment success rates are applicable only to cases with drug-sensitive TB.

**Fig 1:**
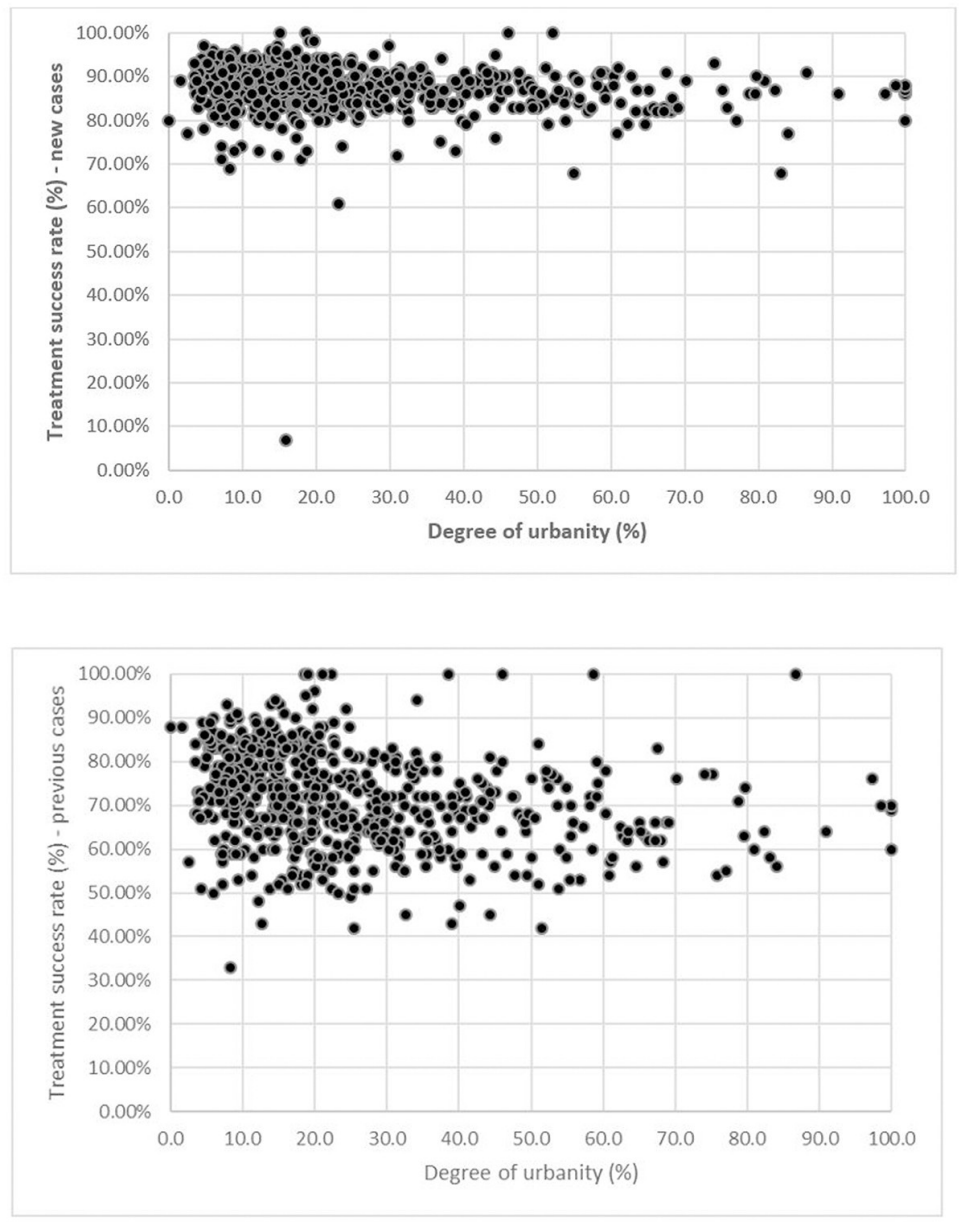
**a.** District-level treatment success rate among new TB cases versus degree of urbanity. **b.** District-level treatment success rate among previously treated cases versus degree of urbanity.

In order to determine how district-level TB treatment outcomes vary with the extent to which a district is urban or rural, we used the degree of urbanity of each district reported in the 2011 decadal census, the most recent available [14]. Note that the district boundaries wherein the RNTCP operates may be different in comparison with the boundaries of administrative districts; however, the TB India reports do not mention any differences between the RNTCP districts and administrative districts, and hence we assume that the RNTCP districts correspond to administrative districts. Plots of district-level treatment success rates among new and previously treated cases versus district-level degree of urbanity are depicted in Fig 1a and 1b below.

From the figures, it appears that there is greater variation in treatment outcomes among areas with lower urbanity. Two methods of formally investigating differences in treatment outcomes based on degree of urbanity suggest themselves: the first involves directly conducting a regression analysis to determine whether a significant non-zero trend exists between treatment outcomes and degree of urbanity; whereas the second involves performing a classification of districts based on degree of urbanity, and comparing treatment outcomes across classes or clusters. We perform both types of analyses, and compare the results from both the direct regression analysis and the cluster-based analysis.

The census data does not provide the classification of districts–for example, into “urban” and “rural”—based on degree of urbanity. Therefore, we applied an unsupervised machine learning method called K-means clustering on the district-level degree of urbanity data to obtain the classification. K-means clustering is used for grouping unlabeled data (that is, data without labels such as ‘urban’, ‘rural’, ‘semi-urban’, etc.) into clusters that are formed based on the degree of similarity between individual data points. Here ‘K’ refers to the number of clusters that the data should be grouped into, which is specified by the analyst prior to applying the method. We chose to apply this method of analyzing how treatment outcomes vary with degree of urbanity, instead of (for example) reporting and analyzing treatment outcomes by groups of districts formed using deciles of degree of urbanity, as K-means clustering determines optimal group/cluster boundaries based on patterns detected in the data without the imposition of artificial grouping rules by the analyst.

A natural approach for the clustering analysis is to specify K = 2, for division into rural and urban clusters; however, we determined that the optimal number of clusters (i.e., the number of clusters that achieve minimal within-cluster variances) is 15. Therefore, we group districts into 15 clusters based on degree of urbanity. Then, we analyze how average treatment success rates among new and previously treated cases vary across clusters. The results of the clustering exercise are summarized in Table 1 below. This includes the average and the coefficient of variation (CV; defined as the ratio of the standard deviation to the mean) of the treatment success rates among new and previously treated cases for each cluster. More details regarding the clustering exercise, including the method by which we determined the optimal number of clusters, are provided in the online supporting information.

**Table 1:** RNTCP treatment outcomes by district cluster.

Cluster	Average degree of urbanity (%)	Number of districts	Average population of district (millions)	Treatment success rate statistics (%)			
				Average, prev. treated cases	Average, new cases	CV, prev. treated cases	CV, new cases
1	4.9	40	1.685	76.5	89.1	12.6	4.8
2	8.4	71	1.659	74.8	87.6	12.1	5.8
3	11.8	55	1.895	73.8	87.9	14.4	5.3
4	15.6	70	1.436	73.2	88.2	14.5	5.4
5	19.8	73	1.786	72.9	88.4	16.6	4.5
6	24.0	57	2.032	70.4	86.9	16.4	4.6
7	29.3	47	2.100	68.2	87.3	11.7	4.0
8	34.3	26	2.402	69.8	87.3	12.4	3.2
9	38.9	24	2.203	67.9	86.0	15.3	4.7
10	43.9	22	2.293	68.6	87.9	16.8	5.8
11	50.7	23	2.399	66.4	86.4	15.9	5.1
12	57.9	19	2.593	67.8	86.3	16.9	6.8
13	66.2	17	3.179	64.9	84.0	9.8	4.2
14	78.5	9	4.031	66.0	86.6	12.1	4.3
15	98.1	7	4.012	60.6	84.7	25.8	4.8

Prev. = previously; CV = coefficient of variation

Before we apply regression to quantify the trends in the average treatment success rates across clusters, we discuss the results of directly applying regression to the treatment outcomes and degree of urbanity data. A simple linear regression analysis was conducted. Breusch-Pagan tests and quantile-quantile plots were used to verify that no heteroscedasticity was present, and that the residuals were normally distributed. The regression analyses reveal significant decreasing trends in treatment success rates with increases in degree of urbanity, with the slopes of both lines significantly different from zero (p-values less than 10^**−5**^). However, the R-squared values for the trend lines are low, likely a consequence of a high degree of variance in treatment success rates. This is in accordance with the relatively high CVs observed in Table 1 for as well. This motivates the regression analysis of trends in the cluster-specific average treatment success rates in Table 1, in that analyzing the averages of the cluster-wise treatment success rates may reveal trends less subject to noise. More details regarding the results of this analysis are provided in the online supporting information.

**Fig 2:**
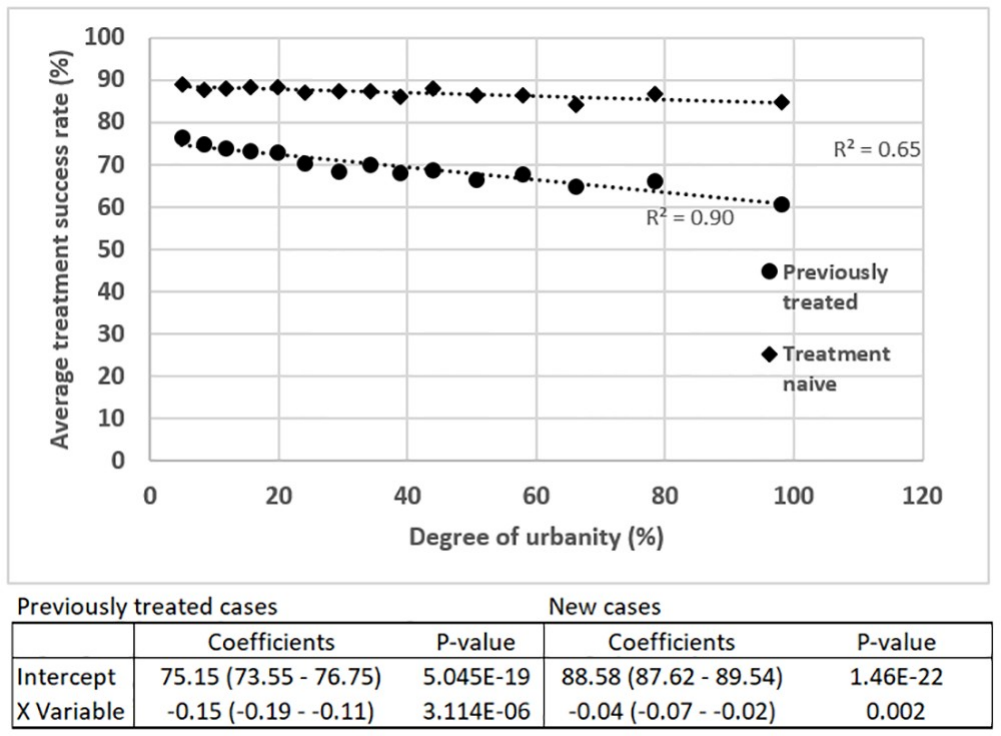
Results of the linear regression analysis between average treatment success rates among new and previously treated cases and degree of urbanity.

The results of the regression analyses on the average treatment success rates are provided in Fig 2 below. Once again, Breusch-Pagan tests and quantile-quantile analyses verified that no heteroscedasticity was present and that the residuals were normally distributed.

The average treatment success rates among new and previously treated cases decrease with an increase in degree of urbanity, with a more substantial rate of decrease observed for previously treated cases. A similar clear trend is not present for the CVs of the treatment success rates among new and previously treated cases. However, some of the highest CVs for both new and previously treated cases are found in clusters which correspond to districts with 40–60% urban population, which can be considered to be in a transition zone between predominantly rural and urban areas. A CV of nearly 26% for previously treated cases is observed in districts that are almost entirely urban.

### Transmission dynamics modeling

In order to determine how these differences in TB treatment outcomes translate into differences in epidemiological outcomes based on degree of urbanity, we utilized a mathematical model of TB transmission dynamics. Instead of developing a *de novo* model, we decided to modify a recently published TB dynamic transmission model in the Indian context, developed by Mandal and colleagues [7]. The model captures the dynamics of diagnosis, treatment and transmission of TB in India, including the scale-up of RNTCP from 1997 onwards. However, the model does not describe the different modes of treatment discontinuation and treatment for previously treated cases. Given that the differences in treatment success rates between clusters are observed to be substantially higher among previously treated cases, we modified the model to include health states for cases seeking treatment again after undergoing one or more previous courses of treatment, so that the differences in outcomes between treatment-naïve and previously treated cases are fully captured. The simplifying assumptions underlying Mandal and colleagues’ model [7] are retained in our version as well: we do not consider distinctions between smear-positive, smear-negative and extra pulmonary TB; and we also do not consider HIV as a comorbidity. Therefore, when using the model to investigate the impact of differences in treatment outcomes between clusters on epidemiological outcomes, we assume that the treatment outcomes for smear-positive cases obtained from the 2014 TB India report [13] apply to the patient population considered in the model as well. We also reiterate that because it is not clear whether the treatment outcomes from the TB India report apply to drug-resistant TB as well, we make the conservative assumption that when the treatment success rates from the report are applied in the model, we assume that it applies only to the drug-sensitive component of the model.

Key health states we added to the model include states corresponding to cases undergoing treatment again after (a) failure of previous treatment; (b) defaulting from previous treatment; (c) relapse of TB after previous treatment; and (d) recurrence of TB after previous treatment. The definitions of treatment failure, treatment default, TB relapse and TB recurrence are consistent with those used in the TB India reports. These states are included for both drug-sensitive (DS) TB as well as multi-drug resistant (MDR) TB. Details regarding patient flow through the model, equations governing the transmission dynamics, and parameter estimation are provided in the online supporting information.

We calibrated our model to prevalence and incidence values (at specific points in time as well as trends over time) from the Mandal study, and from other sources [1,15], including the 2016 WHO TB report, and a meta-analysis of MDR-TB prevalence studies. A more comprehensive approach towards calibration might have been to calibrate the model to a similar set of calibration targets for each cluster, and then use the cluster-specific model along with the cluster-specific treatment success rates among new and previously treated cases to project the effect of differences in treatment outcomes on epidemiological outcomes. However, this would require estimation of cluster-specific calibration targets for–at minimum–TB incidence, prevalence, and notifications. This in turn would require the availability of estimates of TB incidence, prevalence and notifications at district-level resolution. However, even the TB India reports, the most comprehensive source of TB data for India, do not provide such estimates. While TB India reports, especially up to the 2014 report [13], provide district-level estimates of notifications of smear-positive and smear-negative cases, they do not provide district-level estimates of incidence and prevalence. Further, as mentioned earlier, no mention was made in the TB India reports regarding distinctions in the notifications of DS-TB and MDR-TB. Thus, the use of notification rates from the TB India reports would yield only a single calibration target for each cluster. However, we were able to obtain multiple calibration targets at the national level for both DS-TB and MDR-TB at a single point in time (2015) as well as across 1998–2015. Hence we opted to calibrate the model to both cross-sectional and longitudinal estimates of incidence, prevalence, and notifications developed for India in its entirety and use this model for cluster-specific analyses using cluster-specific treatment outcomes data.

**Fig 3:**
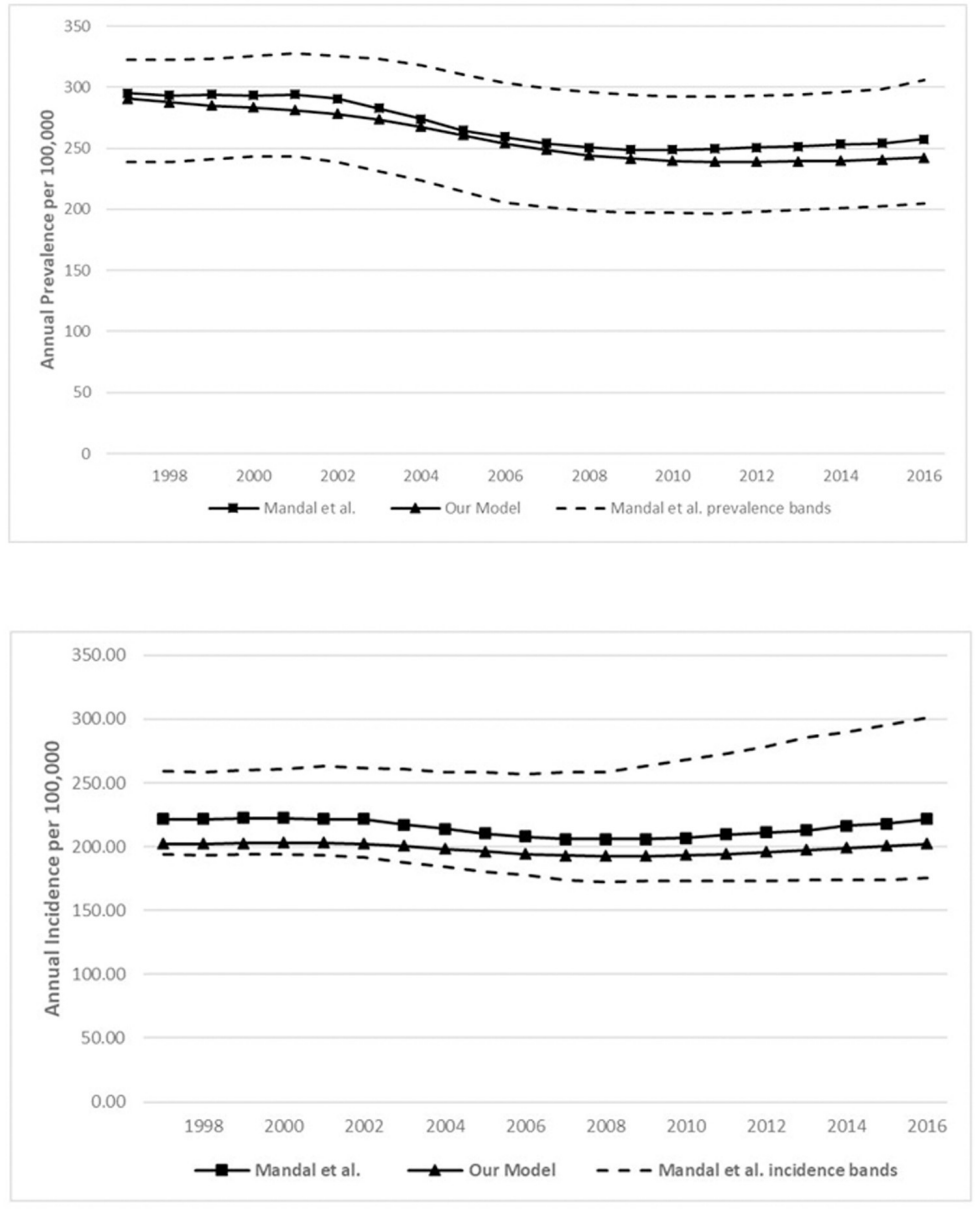
**a.** Comparison between prevalence curves from Mandal and colleagues (2017) and our model. **b.** Comparison between incidence curves from Mandal and colleagues (2017) and our model.

Table 2 provides the calibration targets and the output obtained by our model, and Fig 3a and 3b depicts the prevalence and incidence trends generated by our model compared to those from the Mandal study. Our model performs reasonably well with respect to the calibration targets, and generates outcomes that are well within the uncertainty intervals for both cross-sectional and longitudinal calibration targets.

**Table 2:** Model calibration—Targets and outcomes.

Epidemiological Indicator	Calibration Target	Model output
Incidence in 2015 [1]	217 (112–355)	202
Prevalence in 2015 [7]	253 (195–312)	241
Proportion of MDR cases among newly diagnosed cases: average across 2006–15 [15]	5.6% (3.8–7.4)	5.55%
Proportion of MDR cases among previously treated cases: average across 2006–15 [15]	35.8% (29.2–42.4)	31.3%
TB notifications in 2015 [1]	1.74 million	1.70 million
Cumulative TB notifications, 1997–2015 [7]	19.61 (17.65–21.57) million	20.39 million

## Results

We begin by computing the probability that a TB patient seeking treatment in a district from a given cluster will receive treatment with a success rate below that of the national average. This was computed by modeling the treatment success rates as beta random variables, a common choice for modeling uncertainty around probabilities [16]. The parameters of the beta distributions for each cluster’s treatment success rates were computed using their respective means and standard deviations. The results of this computation are presented in Table 3 below.

**Table 3:** Probabilities of receiving TB treatment with a success rate below the national average.

Cluster	Degree of Urbanity (%)	Probability of receiving treatment with a success rate below the national average (%)	
		Treatment-naïve cases	Previously treated cases
1	4.9	30.4	25.8
2	8.4	42.9	30.6
3	11.8	40.6	35.8
4	15.6	38.2	37.9
5	19.8	35.6	39.4
6	24.0	50.4	47.8
7	29.3	46.8	60.4
8	34.3	47.4	51.8
9	38.9	59.6	58.1
10	43.9	40.6	54.2
11	50.7	56.5	63.5
12	57.9	51.8	57.1
13	66.2	82.3	81.5
14	78.5	54.3	70.9
15	98.1	72.2	71.6

We first note that districts in clusters 11–15 (higher degrees of urbanity) are more likely to provide treatment with success rates below the national average success rate. We infer from Tables 1 and 3 that treatment-naïve cases in nearly 28% of all districts are likely to (i.e., with probability > 0.5) receive treatment with success rates below the national average. These districts contained approximately 35% of the Indian population, according to the 2011 census [14]. Similarly, previously treated cases in 35% of all districts (containing approximately 43% of the Indian population) are likely to receive treatment with success rates below the national average.

**Fig 4:**
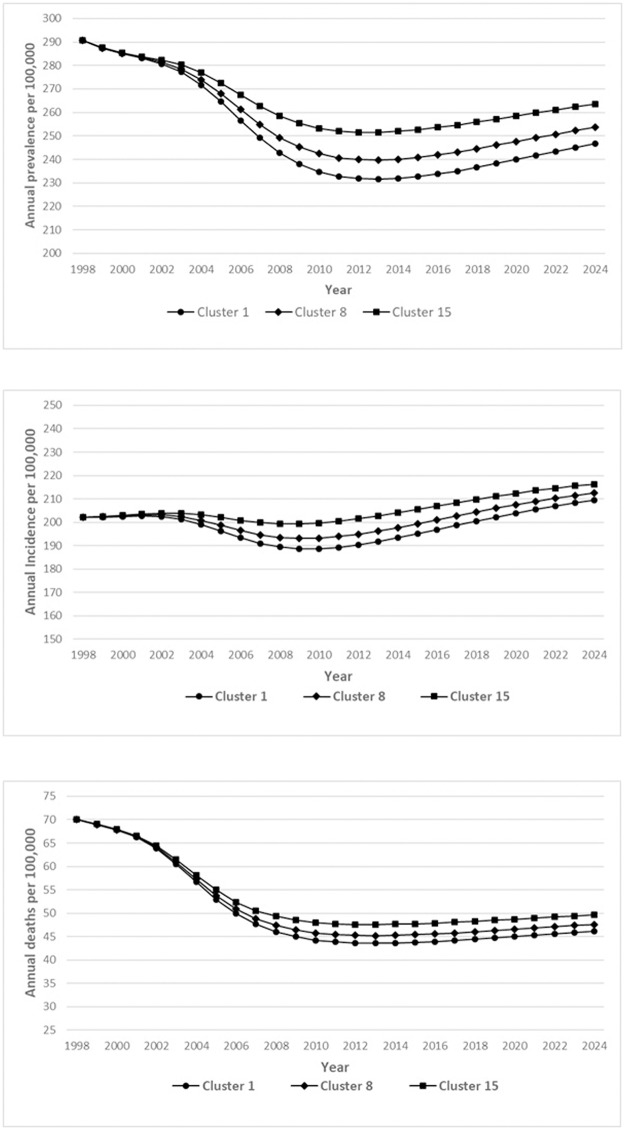
**a.** Prevalence projections for 2019–2024 for best, worst and average performing clusters. **b.** Incidence projections for 2019–2024 for best, worst and average performing clusters. **c.** Mortality projections for 2019–2024 for best, worst and average performing clusters.

Next, we use the clustering results (Table 1) with the model to project the incidence, prevalence and mortality in selected clusters from 2019–2024 (six years). We select three clusters: clusters 1, 8, cluster 15. Clusters 1 and 15, with average urbanities of 4.9% and 98.3%, represent the best and worst performing clusters, with approximately the highest/lowest mean treatment success rates and lowest/highest degrees of urbanity among both new and previously treated cases. Cluster 8, with an average urbanity of 34.3%, was selected to represent a performance close to the national average. The projections are depicted in Fig 4a–4c.

The largest impact due to variation in treatment success rates between clusters is observed for mortality, with cluster 1 having an average of 3.2% fewer deaths per 100,000 population in comparison with cluster 8 across 2019–2024, and cluster 15 having an average of 4.5% more deaths per 100,000 in comparison with cluster 8, and 7.9% in comparison with cluster 1. A similarly large impact is observed for prevalence across 2019–2024, with cluster 1 having on average 3% fewer cases of TB per 100,000 when compared to cluster 8, and cluster 15 having 4.2% more cases per 100,000 than cluster 8, and 7.9% in comparison with cluster 1. Further, districts in cluster 1 have the second lowest average population (with cluster 2 having the lowest). Therefore, this implies a significantly larger absolute TB-related mortality and health and economic burden for districts in other clusters when compared to cluster 1. For example, the average cluster 15 district is projected to have an annual average of 2,463 TB-related deaths across 2019–2024 compared to 1,421 deaths for cluster 8, and 958 deaths per year for an average cluster 1 district.

Finally, using the model, we propagate the cluster-specific information regarding variation in treatment success rates among new and previously treated cases to estimate the uncertainty in epidemiological outcomes such as prevalence, incidence and mortality for each cluster. This is accomplished by utilizing the characterization of the treatment success rates among new and previously treated cases as beta random variables in the transmission model, and conducting a Monte Carlo simulation by sampling from the distributions for these model parameters to estimate the variation around the prevalence, incidence and mortality estimates generated by the model. Note that we do not conduct a full-fledged Monte Carlo simulation for the model that incorporates uncertainty around all model parameters. This is because we are concerned primarily with the effect of variation in the treatment success rates on the variation in epidemiological outcomes, and given that the variation in other model parameters would remain the same across clusters, it is unlikely to affect the relative differences in variation in epidemiological outcomes across clusters. The results of these cluster-wise simulations for one year– 2018 –are provided in Table 4 below, along with the cluster-wise variation in treatment success rates for new and previously treated cases provided for context.

**Table 4:** Propagation of variation in treatment success rates to epidemiological outcomes.

Cluster (% urbanity)	Mean, CV: treatment naïve	Mean, CV: previously-treated	Mean prevalence per 100,000 (95% CI)	Mean incidence per 100,000 (95% CI)	Mean TB-related mortality per 100,000 (95% CI)	Probability > national average
1 (4.9%)	89.1%, 4.7%	76.5%, 12.6%	236.2 (222.6–249.7)	200.3 (193.6–207.0)	44.3 (41.6–47.0)	15.2%
2 (8.4%)	87.6%, 5.8%	74.8%, 12.1%	239.9 (225.6–254.2)	202.1 (195.1–209.1)	45.1 (42.2–47.9)	32.0%
3 (11.8%)	87.9%, 5.3%	73.7%, 14.4%	239.9 (224.4–255.4)	202.1 (194.5–209.7)	45.1 (42.0–48.2)	33.2%
4 (15.6%)	88.2%, 5.4%	73.2%, 14.5%	240.3 (224.5–256.1)	202.3 (194.6–210.0)	45.2 (42.0–48.3)	35.5%
5 (19.7%)	88.4%, 4.5%	72.9%, 16.5%	240.3 (223.8–256.9)	202.3 (194.2–210.4)	45.2 (41.8–48.5)	36.3%
6 (24.0%)	86.9%, 4.6%	70.4%, 16.4%	244.4 (228.1–260.7)	204.3 (196.4–212.1)	46.0 (42.7–49.2)	55.1%
7 (29.3%)	87.3%, 4.0%	68.2%, 11.7%	245.7 (233.3–258.1)	205.0 (199.0–210.9)	46.2 (43.7–48.7)	64.9%
8 (34.3%)	87.3%, 3.2%	69.8%, 12.4%	244.6 (232.1–257.2)	204.4 (198.4–210.5)	46.0 (43.5–48.5)	58.3%
9 (38.9%)	86.0%, 4.7%	67.9%, 15.3%	247.8 (231.8–263.7)	205.9 (198.3–213.5)	46.7 (43.4–49.9)	70.9%
10 (43.9%)	87.9%, 5.8%	68.6%, 16.8%	245.2 (227.1–263.2)	204.6 (196.0–213.3)	46.1 (42.5–49.8)	57.9%
11 (50.7%)	86.4%, 5.1%	66.3%, 15.9%	249.1 (232.3–266.0)	206.6 (198.6–214.6)	46.9 (43.5–50.3)	75.2%
12 (57.9%)	86.3%, 6.8%	67.8%, 16.9%	247.9 (228.8–267.1)	206.0 (196.9–215.1)	46.7 (42.8–50.5)	68.3%
13 (66.2%)	84.1%, 4.2%	64.9%, 9.7%	253.2 (242.3–264.0)	208.5 (203.4–213.7)	47.7 (45.6–49.9)	96.4%
14 (78.5%)	86.6%, 4.3%	66.0%, 12.1%	248.8 (235.8–261.8)	206.4 (200.2–212.7)	46.9 (44.2–49.5)	79.7%
15 (98.1%)	84.7%, 4.8%	60.6%, 25.8%	257.2 (232.5–281.9)	210.3 (198.9–221.7)	48.5 (43.6–53.5)	86.6%
Country	87.3%, 1.2%	70.7%, 2.9%	243.3 (238.8–247.7)	203.8 (201.7–206.0)	45.7 (44.9–46.6)	--

We note that all the 95% confidence intervals around the cluster-specific estimates of prevalence, incidence and mortality in 2018 overlap with those of the national average. However, for districts with higher degree of urbanity (e.g., cluster 6 onwards), the 95% confidence intervals shift further away from that of the country as a whole, with the biasing occurring towards higher values of prevalence, incidence and mortality. In order to quantify this overall upward shift of epidemiological outcomes with increase in degree of urbanity, we calculate the probability of these epidemiological outcomes for a given cluster being greater than the corresponding national averages. For example, for cluster 15, there is an 86.6% probability that a district in the cluster has a TB prevalence, incidence and mortality greater than national average prevalence, incidence or mortality rate per 100,000 population. We calculate a single probability estimate for all three epidemiological outcomes because the same amount of variation around treatment outcomes is propagated to all epidemiological outcomes, and hence the variation around the epidemiological outcomes is identical. This was confirmed by applying the Kruskal Wallis nonparametric test to the normalized data for all epidemiological outcomes.

The probability estimate is highest for cluster 13 (96.4%), significantly higher than the cluster with lowest average treatment success rates and relatively high variation (cluster 15). This is likely because the probability of having a higher incidence, prevalence and mortality rate per 100,000 population than the national average depends not only on the CVs of treatment success rates for the cluster, but also on the average values. The distributions of epidemiological outcomes for cluster 13, with treatment success rates substantially lower than the national average and smaller CVs when compared to other clusters, therefore have very little overlap with the higher side of the national averages for these epidemiological outcomes. This serves to illustrate that improving TB control in these clusters would involve both increasing the average quality and ensuring uniformity in quality of care in each district.

## Discussion

In this study, we investigate the differences in TB outcomes between urban and rural India using district-level treatment outcomes data from the annual TB India reports published by the Central TB Division, Government of India. This is the first study, based on our survey of the literature, which has investigated how TB treatment outcomes vary across districts in conjunction with the proportion of urban population in each district. We also did not identify any other studies in the literature that utilized machine learning methods to perform such an analysis. Only one study directly investigated differences in RNTCP treatment outcomes between urban and rural areas [17]. The authors investigated differences in RNTCP indicators between cases treated in an urban TB unit and in a rural TB unit in West Bengal, a northeastern Indian state. They found statistically significant differences only in failure rates (urban failure rates were higher) between the urban and rural unit. Given that this study was limited to only two TB units in a single state, the need for an analysis of differences in TB treatment outcomes and epidemiology between urban and rural areas across the country is clearly evident. One other study [18] investigated the socioeconomic impact of TB on cases and their families and found that the economic impact in rural areas was significantly lower than in urban areas; however, they authors did not consider differences in TB treatment outcomes or epidemiology.

Even in the international context, a limited number of studies appear to have been conducted to investigate differences in TB treatment outcomes. Key studies include one in the United Kingdom [19], Southern Ethiopia [20], and the Solomon Islands [21]. However, none of these examine differences in epidemiological outcomes beyond TB notifications and treatment outcomes, and neither do they use transmission dynamics models to estimate these outcomes. Further, these studies use binary classifications of regions or treatment units as being urban and rural, whereas we directly use the proportion of urban population in a district in our analysis. Our study provides a proof of concept for the analysis of TB treatment outcomes that can be applied to other settings with a high TB burden and urban-rural inequities.

The majority of the international and Indian studies cited above find that treatment outcomes are either better or are not statistically worse in urban areas when compared to rural areas. Our analysis of the RNTCP treatment outcomes data indicates the presence of trends in the opposite direction–our linear regression analyses find that average treatment success rates among both and new previously treated cases decrease with increase in the proportion of urban population. Further, we find that the highest values of within-cluster variation in treatment success rates also occur in clusters with higher degrees of urbanity. In order to verify that these results were not anomalous, we conducted a linear regression analysis with district-level treatment success rates among new and previously treated cases obtained from the 2011 TB India report (an analysis similar to that described in Section 2) and found a similar, albeit less steep, decrease in treatment outcomes with increasing degree of urbanity. Potential reasons for the decrease in treatment success rates with increasing urban populations could involve difficulties in ensuring quality of care and prevention of infection and transmission in densely populated areas with large disparities in sanitation and access to health care. Predominantly rural areas on the other hand may have a single source of TB treatment under RNTCP with a relatively lower burden per TB unit. This may lead to uniformly better treatment quality as well as surveillance under RNTCP. We hope that the results from our study may motivate formal investigation into the causes of these disparities and the development of methods to address these disparities.

Our analysis is based on data from the 2014 TB India report. Later reports–the most recent being the 2018 TB India [22]–do not provide treatment outcomes data at the district level; hence we were unable to use more recent data in our analysis. This is a limitation of our analysis, and thus illustrates the need for the publication of recent district-level RNTCP treatment outcomes data. In addition to treatment outcomes, the 2014 TB Indian report contains more district-level data regarding RNTCP indicators; for example, it contains smear positive, smear negative and extra-pulmonary case notification rates, proportion of TB cases with an HIV coinfection, etc. The scope of this study is limited to urban-rural disparities in treatment success rates among new and previously treated cases; however, future avenues of research can include a comprehensive analysis of this data to reveal disparities between urban and rural areas in any other RNTCP indicators.

The results generated using the dynamic transmission model indicate that the between-cluster differences in treatment outcomes are likely to translate into significant differences in epidemiological outcomes such as prevalence, incidence and TB-related mortality. A key insight generated by the model is that a cluster or a region with low average treatment success rates as well as low variation in treatment success rates (e.g., cluster 13) is more likely to have epidemiological outcomes worse than the national average than when compared to a cluster or region with lower average treatment success rates and higher variation when compared to the cluster in consideration (e.g., cluster 15 when compared to cluster 13). This is because the cluster that is ostensibly performing worse owing to its lower average treatment success rate and high variation (implying greater inconsistency in treatment quality) benefits from the high treatment success rates that contribute to its high variation in comparison with the cluster that is ostensibly better performing only offers low treatment success rates with little variation. This in turn implies that improving TB treatment under RNTCP even in specific pockets can improve epidemiological outcomes as a whole for a region.

The mathematical model we have adapted as part of this study provides a comprehensive accounting of the transmission dynamics of both drug-sensitive and multi-drug resistant TB. We anticipate that this model structure can be used in multiple other applications, such as to assess the cost-effectiveness of a screening programme targeted at high-prevalence regions. A key challenge in developing such a model in the Indian context is the lack of data for certain parameters, especially for the non-RNTCP components of the model. While the model generates outcomes that statistically conform to epidemiological calibration targets, we anticipate that the model will require updating when data for the parameters becomes available. As explained in a previous section, this limitation extends to model calibration as well, and therefore an avenue for future work involves the use of district-level estimates of incidence, prevalence and notifications as cluster-specific calibration targets for models. The need for such data is further supported by the publication of a relatively recent study [23], which estimated that urban TB cases infect more individuals per year when compared to rural cases, but remain infectious for a significantly shorter duration than rural cases (one year compared to two years).

Overall, we believe our study provides a window into the disparities between urban and rural areas in terms of TB treatment outcomes in the Indian context. Our use of a dynamic transmission model to propagate differences in treatment success rates to differences in epidemiological outcomes provides a proof of concept for the use of a model in estimating the effect of such disparities in TB treatment outcomes on the prevalence, incidence and mortality of tuberculosis in similar settings with a high burden of disease.

## Supporting information

S1 Fig **a.** District-level treatment success rate among new cases versus degree of urbanity: regression results. **b.** District-level treatment success rate among previously treated cases versus degree of urbanity: regression results.
(PNG)

S2 Fig Elbow test results: Change in clustering sum of squared errors with number of clusters. (JPG)

S3 Fig **a.** Model health states and patient flow: drug-sensitive TB. **b.** Model health states and patient flow: multi-drug resistant TB.
(JPG)

S1 Table TB transmission dynamics model parameters. (PDF)
