# Supplementary figures and images for: A model-based investigation into urban-rural disparities in tuberculosis treatment outcomes under the Revised National Tuberculosis Control Programme in India
Source: PLoS One. 2020 Feb 14;15(2):e0228712. doi: 10.1371/journal.pone.0228712 (PMC7021308; doi:10.1371/journal.pone.0228712)

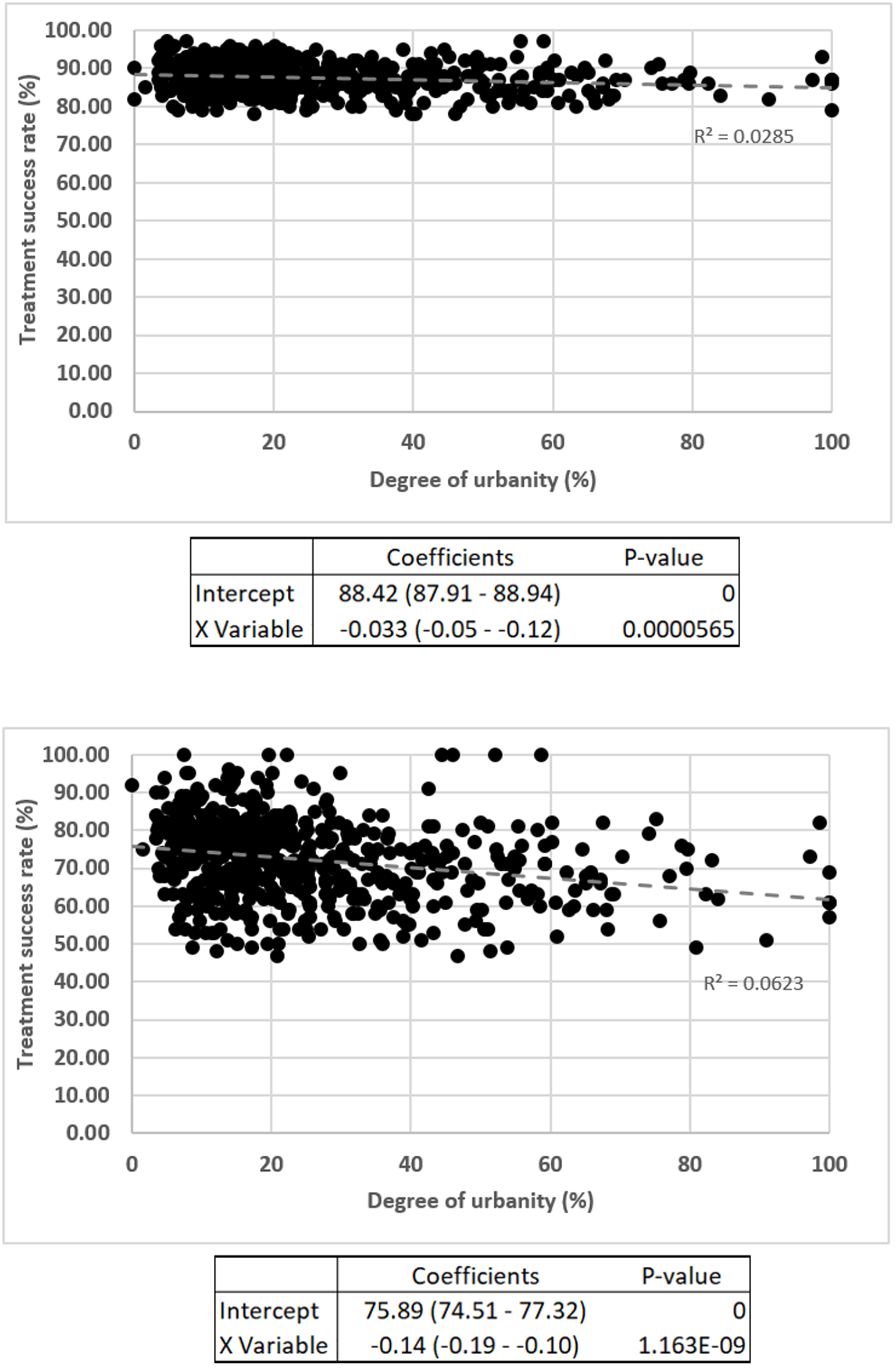

Supplement: S1 Fig — a. District-level treatment success rate among new cases versus degree of urbanity: regression results. b. District-level treatment success rate among previously treated cases versus degree of urbanity: regression results. (PNG) [file pone.0228712.s001.PNG]

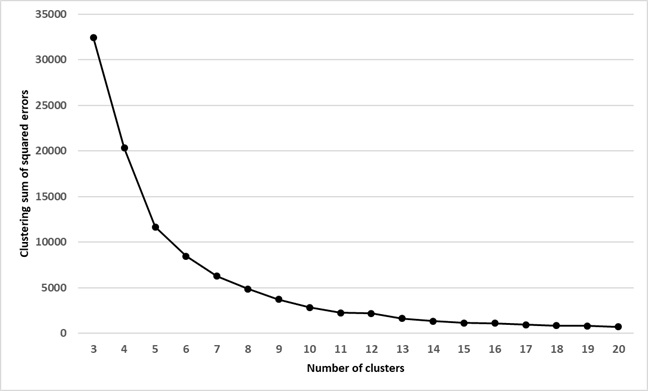

Supplement: S2 Fig — (JPG) [file pone.0228712.s002.jpg]

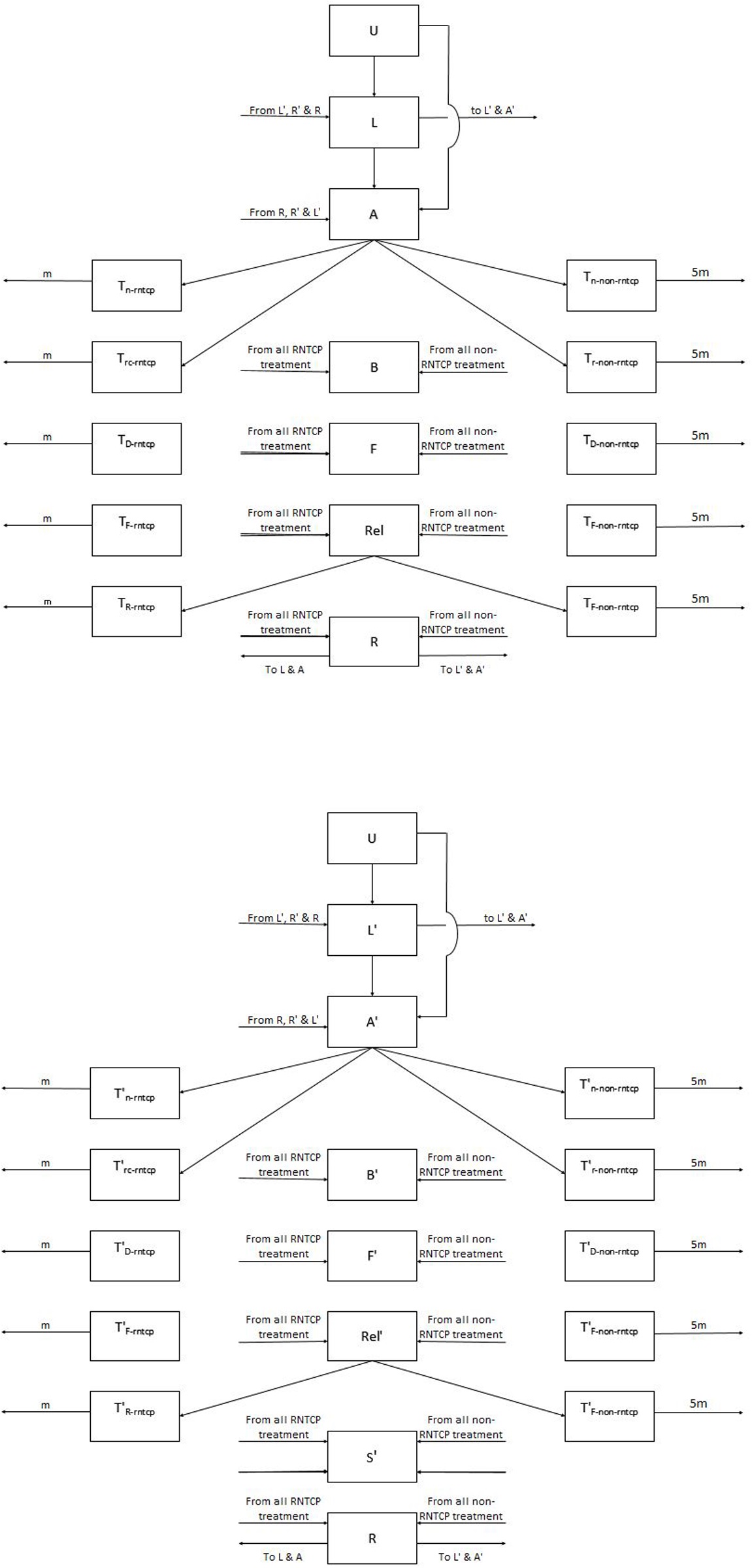

Supplement: S3 Fig — a. Model health states and patient flow: drug-sensitive TB. b. Model health states and patient flow: multi-drug resistant TB. (JPG) [file pone.0228712.s003.JPG]
