# Supplementary material for: A model-based investigation into urban-rural disparities in tuberculosis treatment outcomes under the Revised National Tuberculosis Control Programme in India
Source: PLoS One. 2020 Feb 14;15(2):e0228712. doi: 10.1371/journal.pone.0228712 (PMC7021308; doi:10.1371/journal.pone.0228712)
Supplement: S1 Table — (PDF) [file pone.0228712.s004.pdf]

PLoS One

A Model-based Investigation into Urban-Rural Disparities in Tuberculosis Treatment Outcomes under the Revised National Tuberculosis Control Programme in India

Himanshu Singh, Varun Ramamohan

Supporting Information File

Direct Regression Results: Treatment Success Rates versus Degree of Urbanity

In this section, we present more details regarding the regression conducted between the treatment success rates among new and previously treated cases and degree of urbanity without performing any clustering of the data. The results are summarized in Figures S1(a) and S1(b) below.

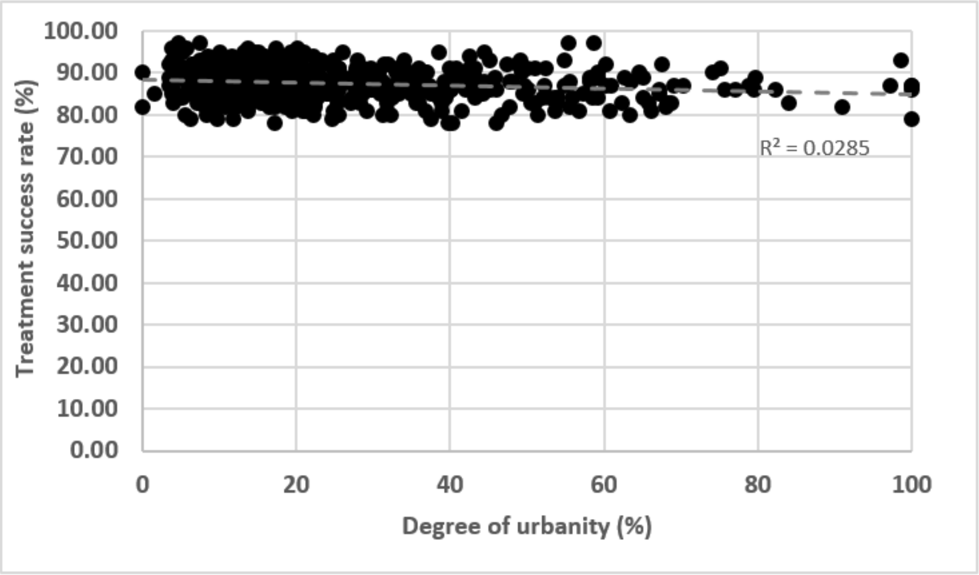

	Coefficients	P-value
Intercept	88.42 (87.91 - 88.94)	0
X Variable	-0.033 (-0.05 - -0.12)	0.0000565

Figure S1(a). District-level treatment success rate among new cases versus degree of urbanity: regression results.

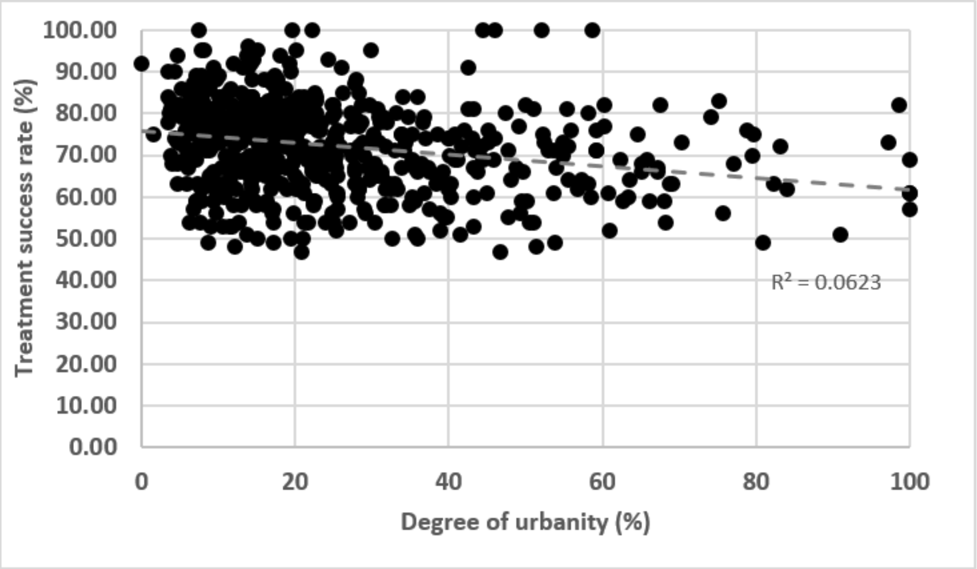

	Coefficients	P-value
Intercept	75.89 (74.51 - 77.32)	0
X Variable	-0.14 (-0.19 - -0.10)	1.163E-09

Figure S2(a). District-level treatment success rate among previously treated cases versus degree of urbanity: regression results.

K-means Clustering

The method divides data into a pre-specified number of clusters (denoted by K) by determining the distance of each data point from the mean of each cluster. The cluster means are randomly initialized before the clustering begins, and are updated with each iteration – that is, when each data point is placed into its nearest cluster. The optimum number of clusters were determined by using the elbow method. The elbow method involves running the clustering technique for different values of K, and calculating the sum of squared errors (SSE) for each value of K. The SSE for each value of K is calculated as the sum of the squared differences between each data point and the mean of the cluster in which it belongs. The SSE is plotted against K and from the plot we choose the value of K that corresponds to the point when the decrease in SSE stops

exceeding 10%. Figure S2 below depicts the use of the elbow method to determine the optimal number of clusters.

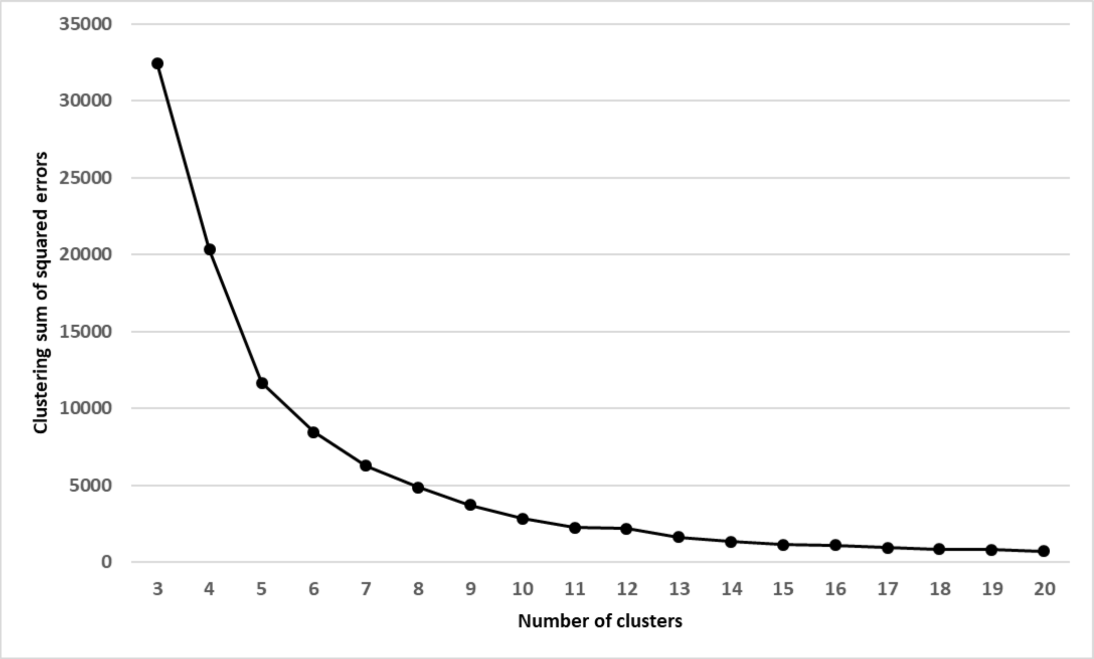

Figure S2. Elbow test results: change in clustering sum of squared errors with number of clusters.

Dynamic Transmission Model Description

The model comprises two parts, corresponding to drug-sensitive TB (Figure S2a) and multi-drug resistant (MDR) TB (Figure S2b). The compartments in the model represent various states of disease and care seeking.

<u>Drug-sensitive TB</u>:

Uninfected individuals (U), after acquiring a new infection will either enter the state of latent infection (L) or can develop pre-treatment active disease (A). Treatment-naïve individuals with active infection start TB treatment with a delay *r*. Treatment can either be initiated under RNTCP services ($T_{n\text{-RNTCP}}$) or under non-RNTCP services ($T_{n\text{-non-RNTCP}}$) for new patients. Patients under

both RNTCP and non-RNTCP services who default or for whom treatment fails move to compartments B and F respectively. We assume these patients will subsequently seek care again after a delay period (*w* and *f*, respectively). As the patients who default and move to B have already sought treatment once and are seeking treatment again, they will move to states designated specifically for previously treated patients who have defaulted. Such states are created for both RNTCP services ($T_{D\text{-}RNTCP}$) and non-RNTCP services ($T_{D\text{-}non\text{-}RNTCP}$). Similarly, patients for whom treatment fails (that is, patients in F) may also seek treatment again under RNTCP services ($T_{F\text{-}RNTCP}$) or under non-RNTCP services ($T_{F\text{-}non\text{-}RNTCP}$). Patients for whom the TB infection relapses after completing a course of treatment seek treatment after a delay *v*. These patients spend the period between treatment completion and re-treatment after relapse in a holding state denoted by *Rel*. They also may receive treatment under RNTCP services ($T_{R\text{-}RNTCP}$) or under non-RNTCP services ($T_{R\text{-}non\text{-}RNTCP}$). Patients whose infection recurs either due to reactivation of a latent infection or due to reinfection move to A and will seek treatment after a delay period (e). These patients are treated in the $T_{rc\text{-}RNTCP}$ or $T_{rc\text{-}non\text{-}RNTCP}$ states. We associate a per-capita TB mortality rate with each of these states. Individuals may be cured (R) either through treatment or spontaneous clearance of their disease. Other aspects of Mandal and colleagues' [1] model, such as movement to and from the DS-TB and MDR-TB states, are also retained in our model. Note that recurrence and reinfection are treated for the most part as a treatment-naïve case.

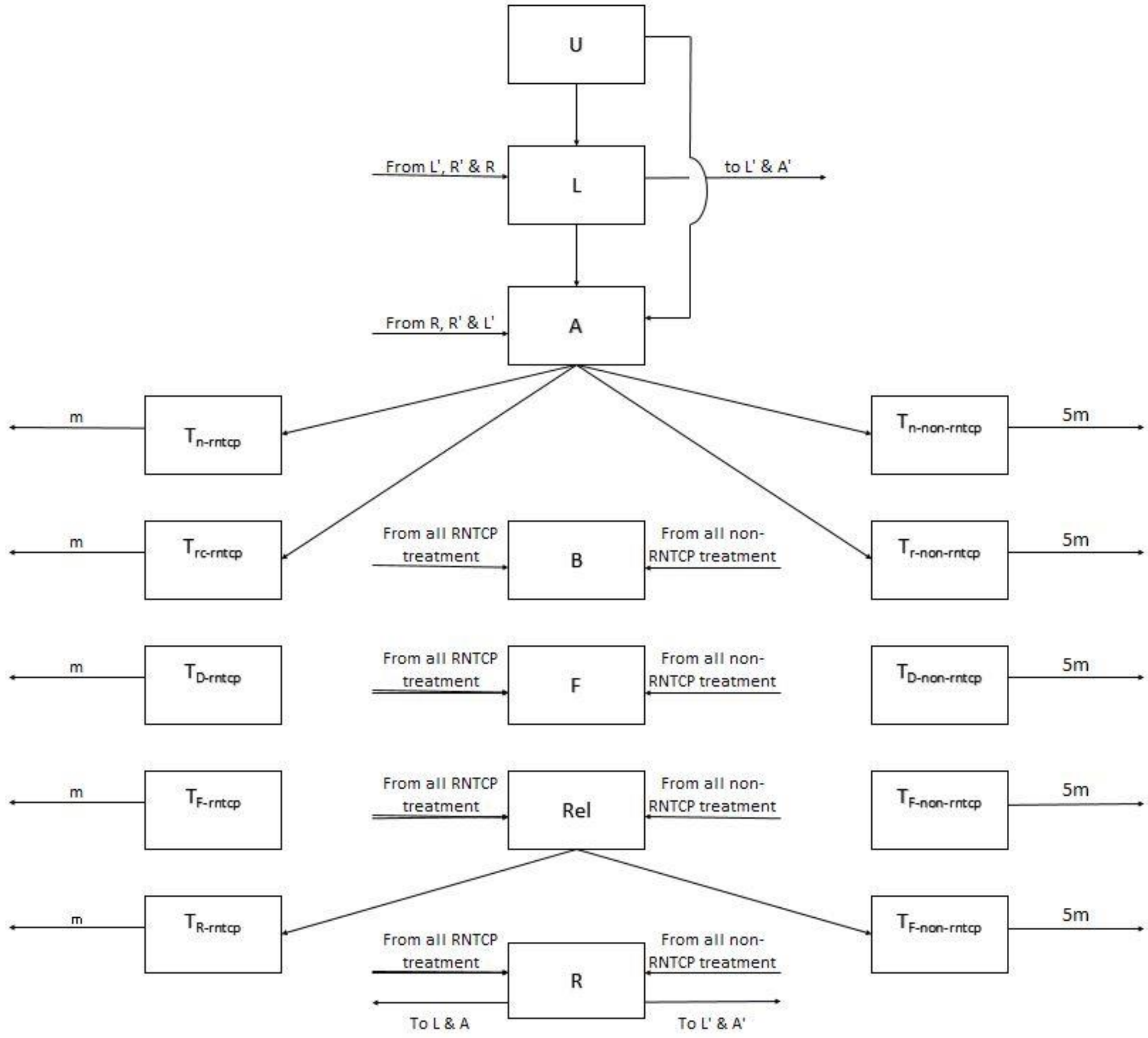

Figure S3(a). Model health states and patient flow: drug-sensitive TB.

<u>Multi-drug resistant TB</u>

The health states and transmission dynamics for MDR-TB are similar to that for DS-TB and hence the associated notation is similar as well. The key difference between the treatment of DS-TB and MDR-TB as captured by the model involves the availability of second-line treatment for patients for whom first-line treatment for MDR-TB fails. Second-line treatment is available only under the

aegis of RNTCP (state S'_{RNTCP}). The compartment S'_{RNTCP} denotes the MDR-TB patients who are receiving second-line treatment only available under RNTCP.

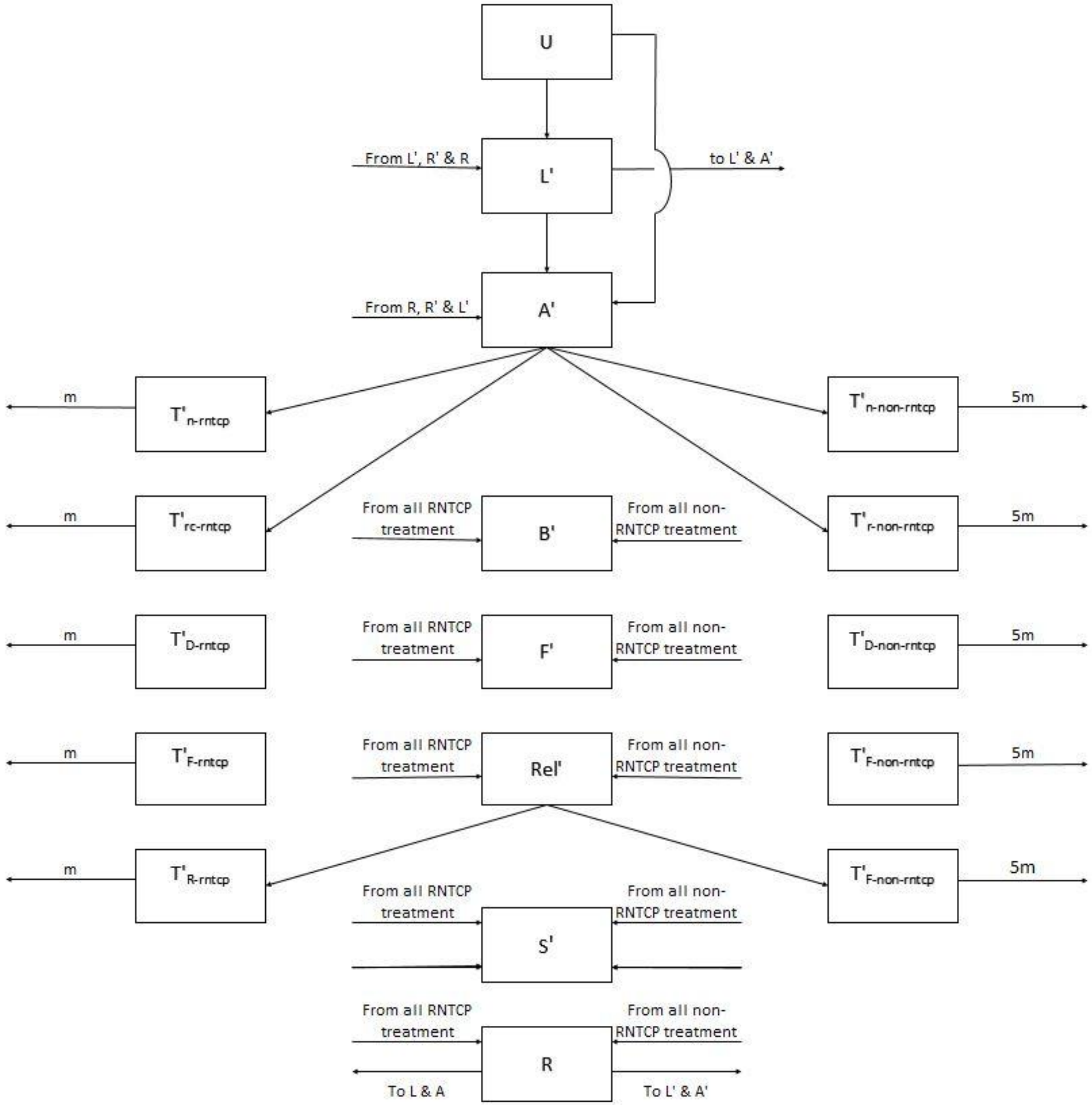

Figure S3(b). Model health states and patient flow: multi-drug resistant TB.

Model Equations

DS-TB section

$$\dot{U} = b - (\lambda + \lambda_{mdr})U$$

$$\dot{L} = (1-k)\lambda U + (1-k)c\lambda(R+R') - hL - c\lambda_{mdr}L - \mu_0 L$$

$$\dot{A} = k\lambda U + hL + (kc\lambda + \rho)R + kc\lambda(R') - (r+e+\sigma)A - \mu_{UTB} A$$

$$\dot{T}_{n-RNTCP} = r\, p(t)\; A - [\tau_{FL} + d_{n-RNTCP} + m + \sigma]T_{n-RNTCP} - \mu_{RNTCP}T_{n-RNTCP}$$

$$\dot{T}_{R-RNTCP} = v\, p(t)\operatorname{Re}l - [\tau_{FL} + d_{R-RNTCP} + n]T_{R-RNTCP} - \mu_R T_{R-RNTCP}$$

$$\dot{T}_{rc-RNTCP} = e\, p(t)A - [\tau_{FL} + d_{rc-RNTCP} + m + \sigma]T_{rc-RNTCP} - \mu_{rc}T_{rc-RNTCP}$$

$$\dot{T}_{D-RNTCP} = w\, p(t)B - [\tau_{FL} + d_{D-RNTCP} + n + \sigma]T_{D-RNTCP} - \mu_D T_{D-RNTCP}$$

$$\dot{T}_{F-RNTCP} = f\; p(t)F - [\tau_{FL} + d_{F-RNTCP} + n]T_{F-RNTCP} - \mu_{F-RNTCP}T_{F-RNTCP}$$

$$\dot{T}_{n-non-RNTCP} = [1 - p(t)]r\, A - [\tau_{FL} + d_{n-non-RNTCP} + 5m + \sigma]T_{n-non-RNTCP} - \mu_{non-RNTCP}T_{n-non-RNTCP}$$

$$\dot{T}_{R-non-RNTCP} = v[1 - p(t)]\operatorname{Re}l - [\tau_{FL} + d_{R-non} + 5n]T_{R-non-RNTCP} - \mu_{R-non}T_{R-non-RNTCP}$$

$$\dot{T}_{rc-non-RNTCP} = e[(1 - p(t)]A - [\tau_{FL} + d_{rc-non} + 5m + \sigma]T_{rc-non-RNTCP} - \mu_{rc-non}T_{rc-non-RNTCP}$$

$$\dot{T}_{D-non-RNTCP} = w[(1 - p(t)]B - [\tau_{FL} + d_{D-non} + 5n + \sigma]T_{D-non-RNTCP} - \mu_{D-non}T_{D-non-RNTCP}$$

$$\dot{T}_{F-non-RNTCP} = f[(1 - p(t)]F - [\tau_{FL} + d_{F-non} + 5n]T_{F-non-RNTCP} - \mu_{F-non}T_{F-non-RNTCP}$$

$$\dot{\operatorname{Re}}l = z\;(\alpha_{pub}\tau_{FL}T_{n-RNTCP} + \alpha_R\tau_{FL}T_{R-RNTCP} + \alpha_{rc}\tau_{FL}T_{rc-RNTCP} + \alpha_d\tau_{FL}T_{d-RNTCP} + \alpha_F\tau_{FL}T_{F-RNTCP} + \alpha_{prv}\tau_{FL}T_{n-non-RNTCP} + \alpha_{R-non}\tau_{FL}T_{R-non-RNTCP} + \alpha_{rc-non}\tau_{FL}T_{rc-non-RNTCP} + \alpha_{d-non}\tau_{FL}T_{d-non-RNTCP} + \alpha_{F-non}\tau_{FL}T_{F-non-RNTCP}\;) - v\operatorname{Re}l - \mu_{TB}\operatorname{Re}l$$

$$\dot{B} = [d_{n-RNTCP}T_{n-RNTCP} + d_{R-RNTCP}T_{R-RNTCP} + d_{rc-RNTCP}T_{rc-RNTCP} + d_{D-RNTCP}T_{D-RNTCP} + d_{F-RNTCP}T_{F-RNTCP} + d_{n-non}T_{n-non-RNTCP} + d_{R-non}T_{R-non-RNTCP} + d_{rc-non}T_{rc-non-RNTCP} + d_{D-non}T_{D-non-RNTCP} + d_{F-non}T_{F-non-RNTCP}] - (w+\sigma)B - \mu_B B$$

$$F\cdot = \tau_{FL}[(1-\alpha_{pub})T_{n-RNTCP} + (1-\alpha_R)T_{R-RNTCP} + (1-\alpha_{rc})T_{rc-RNTCP} + (1-\alpha_D)T_{D-RNTCP} + (1-\alpha_F)T_{F-RNTCP} + (1-\alpha_{prv})T_{n-non-RNTCP} + (1-\alpha_{R-non})T_{R-non-RNTCP} + (1-\alpha_{rc-non})T_{rc-non-RNTCP} + (1-\alpha_{D-non})T_{D-non-RNTCP} + (1-\alpha_{F-non})T_{F-non-RNTCP}] - f\,F - \mu_F F$$

$$\dot{R} = (1-z)(\alpha_{pub}\tau_{FL}T_{n-RNTCP} + \alpha_R\tau_{FL}T_{R-RNTCP} + \alpha_{rc}\tau_{FL}T_{rc-RNTCP} + \alpha_d\tau_{FL}T_{d-RNTCP} + \alpha_F\tau_{FL}T_{F-RNTCP} + \alpha_{prv}\tau_{FL}T_{n-non-RNTCP} + \alpha_{R-non}\tau_{FL}T_{R-non-RNTCP} + \alpha_{rc-non}\tau_{FL}T_{rc-non-RNTCP} + \alpha_{d-non}\tau_{FL}T_{d-non-RNTCP} + \alpha_{F-non}\tau_{FL}T_{F-non-RNTCP}) + \sigma(A + T_{n-RNTCP} + T_{rc-RNTCP} + T_{D-RNTCP} + T_{n-non-RNTCP} + T_{rc-non-RNTCP} + T_{D-non-RNTCP} + B) - (\rho + c\lambda + c\lambda_{mdr})R - \mu_0 R$$

<u>MDR-TB section</u>

$$\dot{L}' = (1-k)\lambda_{mdr}U + (1-k)c\lambda_{mdr}(R+R') - hL' - c\lambda L' - \mu_0 L'$$

$$\dot{A}' = k\lambda_{mdr}U + hL' + (kc\lambda_{mdr} + \rho)R' + kc\lambda_{mdr}(R) - (r+e+\sigma)A' - \mu_{UTB}A'$$

$$\dot{T}'_{n-RNTCP} = p(t)\,r\,A' + mT_{n-RNTCP} - [\tau_{FL} + d'_{n-RNTCP} + \sigma]T'_{n-RNTCP} - \mu'_{RNTCP}T'_{n-RNTCP}$$

$$\dot{T}'_{R-RNTCP} = p(t)\,v\,\mathrm{Re}l' + nT_{R-RNTCP} - [\tau_{FL} + d'_R]T'_{R-RNTCP} - \mu'_R T'_{R-RNTCP}$$

$$\dot{T}'_{rc-RNTCP} = p(t)\,e\,A' + mT_{rc-RNTCP} - [\tau_{FL} + d'_{rc} + \sigma]T'_{rc-RNTCP} - \mu'_{rc}T'_{rc-RNTCP}$$

$$\dot{T}'_{D-RNTCP} = p(t)\,w\,B' + nT_{D-RNTCP} - [\tau_{FL} + d'_D + \sigma)]T'_{D-RNTCP} - \mu'_D T'_{D-RNTCP}$$

$$\dot{T}'_{F-RNTCP} = p(t)[1-q(t)]\,f\,F' + nT_{F-RNTCP} - [\tau_{FL} + d'_F]T'_{F-RNTCP} - \mu'_{F-RNTCP}T'_{F-RNTCP}$$

$$\dot{T}'_{n-non-RNTCP} = [1-p(t)]\,r\,A' + 5mT_{n-non-RNTCP} - [\tau_{FL} + d'_{n-non-RNTCP} + \sigma]T'_{n-non-RNTCP} - \mu'_{non-RNTCP}T'_{n-non-RNTCP}$$

$$\dot{T}'_{R-non-RNTCP} = [1-p(t)]\,v\,\mathrm{Re}l' + 5nT_{R-non-RNTCP} - [\tau_{FL} + d'_{R-non}]T'_{R-non-RNTCP} - \mu'_{R-non}T'_{R-non-RNTCP}$$

$$\dot{T}'_{rc-non-RNTCP} = [1-p(t)]\,e\,A' + 5mT_{rc-non-RNTCP} - [\tau_{FL} + d'_{rc-non} + \sigma]T'_{rc-non-RNTCP} - \mu'_{rc-non}T'_{rc-non-RNTCP}$$

$$\dot{T}'_{D-non-RNTCP} = [1-p(t)]\,w\,B' + 5nT_{D-non-RNTCP} - [\tau_{FL} + d'_{D-non} + \sigma]T'_{D-non-RNTCP} - \mu'_{D-non}T'_{D-non-RNTCP}$$

$$\dot{T}'_{F-non-RNTCP} = [1-p(t)]\,f\,F' + 5nT_{F-non-RNTCP} - [\tau_{FL} + d'_{F-non}]T'_{F-non-RNTCP} - \mu'_{F-non}T'_{F-non-RNTCP}$$

$$\dot{\mathrm{Re}}l' = z\,(\alpha'_{pub}\tau_{FL}T'_{n-RNTCP} + \alpha'_R\tau_{FL}T'_{R-RNTCP} + \alpha'_{rc}\tau_{FL}T'_{rc-RNTCP} + \alpha'_D\tau_{FL}T'_{D-RNTCP} + \alpha'_F\tau_{FL}T'_{F-RNTCP} + \alpha'_{prv}\tau_{FL}T'_{n-non-RNTCP} + \alpha'_{R-non}\tau_{FL}T'_{R-non-RNTCP} + \alpha'_{rc-non}\tau_{FL}T'_{rc-non-RNTCP} + \alpha'_{D-non}\tau_{FL}T'_{D-non-RNTCP} + \alpha'_{F-non}\tau_{FL}T'_{F-non-RNTCP}) - v\,\mathrm{Re}l' - \mu_{TB}\,\mathrm{Re}l'$$

$$\dot{B}' = [d'_{n-RNTCP}\,T'_{n-RNTCP} + d'_{R-RNTCP}\,T'_{R-RNTCP} + d'_{rc-RNTCP}\,T'_{rc-RNTCP} + d'_{D-RNTCP}\,T'_{D-RNTCP} + d'_{F-RNTCP}\,T'_{F-RNTCP} + d'_{n-non-RNTCP}\,T'_{n-non-RNTCP} + d'_{R-non-RNTCP}\,T'_{R-non-RNTCP} + d'_{rc-non-RNTCP}\,T'_{rc-non-RNTCP} + d'_{D-non-RNTCP}\,T'_{D-non-RNTCP} + d'_{F-non-RNTCP}\,T'_{F-non-RNTCP}] - (w+\sigma)B' - \mu_B B'$$

$$F' = (1-\alpha'_{pub})[1-q(t)]\tau_{FL}T'_{n-RNTCP} + (1-\alpha'_R)[1-q(t)]\tau_{FL}T'_{R-RNTCP} + (1-\alpha'_{rc})[1-q(t)]\tau_{FL}T'_{rc-RNTCP} + (1-\alpha'_D)[1-q(t)]\tau_{FL}T'_{D-RNTCP} + (1-\alpha'_F)[1-q(t)]\tau_{FL}T'_{F-RNTCP} + (1-\alpha'_{prv})\tau_{FL}T'_{n-non-RNTCP} + (1-\alpha'_{R-non})\tau_{FL}T'_{R-non-RNTCP} + (1-\alpha'_{rc-non})\tau_{FL}T'_{rc-non-RNTCP} + (1-\alpha'_{D-non})\tau_{FL}T'_{D-non-RNTCP} + (1-\alpha'_{F-non})\tau_{FL}T'_{F-non-RNTCP} - f\,F' - \mu_F\,F'$$

$$\dot{S}'_{RNTCP} = q(t)(1-\alpha'_{pub})\,\tau_{FL}T'_{n-RNTCP} + q(t)(1-\alpha'_R)\,\tau_{FL}T'_{R-RNTCP} + q(t)(1-\alpha'_{rc})\,\tau_{FL}T'_{rc-RNTCP} + q(t)(1-\alpha'_D)\,\tau_{FL}T'_{d-RNTCP} + q(t)(1-\alpha'_F)\,\tau_{FL}T'_{F-RNTCP} + f\,p(t)\,q(t)\,F' + v\,\mathrm{Re}l_S' + wB_S' + fF_S' - (\tau_{SL} + d_S')S'_{RNTCP} - \mu'_S\,S'$$

$$\dot{\mathrm{Re}}l_S' = z\ \alpha'_{pub2}\,\tau_{SL}S'_{RNTCP} - v\,\mathrm{Re}l_S' - \mu_{TB}\,\mathrm{Re}l_S'$$

$$\dot{B}_S' = d_S'S'_{RNTCP} - wB_S' - \mu_{TB}B_S'$$

$$\dot{F}_S' = (1-\alpha'_{pub2})\tau_{SL}S'_{RNTCP} - fF_S' - \mu_F F_S'$$

$$\dot{R}' = (1-z)\ (\alpha'_{pub}\,\tau_{FL}T'_{n-RNTCP} + \alpha'_R\,\tau_{FL}T'_{R-RNTCP} + \alpha'_{rc}\,\tau_{FL}T'_{rc-RNTCP} + \alpha'_D\,\tau_{FL}T'_{D-RNTCP} + \alpha'_F\,\tau_{FL}T'_{F-RNTCP} + \alpha'_{pub2}\,\tau_{SL}S'_{RNTCP} + \alpha'_{prv}\,\tau_{FL}T'_{n-non-RNTCP} + \alpha'_{R-non}\,\tau_{FL}T'_{R-non-RNTCP} + \alpha'_{rc-non}\,\tau_{FL}T'_{rc-non-RNTCP} + \alpha'_{D-non}\,\tau_{FL}T'_{D-non-RNTCP} + \alpha'_{F-non}\,\tau_{FL}T'_{F-non-RNTCP}) + \sigma(A' + T'_{n-RNTCP} + T'_{rc-RNTCP} + T'_{D-RNTCP} + T'_{n-non-RNTCP} + T'_{rc-non-RNTCP} + T'_{D-non-RNTCP} + B') - (\rho + c\lambda + c\lambda_{mdr})R' - \mu_0\,R'$$

With,

$$\lambda = \beta(A + T_{n-RNTCP} + T_{R-RNTCP} + T_{rc-RNTCP} + T_{D-RNTCP} + T_{F-RNTCP} + T_{n-non-RNTCP} + T_{R-non-RNTCP} + T_{rc-non-RNTCP} + T_{D-non-RNTCP} + T_{F-non-RNTCP} + \mathrm{Re}l + B + F)/N$$

$$\lambda_{mdr} = \beta_{mdr}\,(A' + T'_{n-RNTCP} + T'_{R-RNTCP} + T'_{rc-RNTCP} + T'_{D-RNTCP} + T'_{F-RNTCP} + T'_{n-non-RNTCP} + T'_{R-non-RNTCP} + T'_{rc-non-RNTCP} + T'_{D-non-RNTCP} + T'_{F-non-RNTCP} + S'_{RNTCP} + \mathrm{Re}l' + F' + B' + \mathrm{Re}l_S' + F_S' + B_S')/N$$

Model Parameter Estimation

Many model parameter estimates were based on those in the model from Mandal and colleagues [1]. Certain parameters, such as the average annual numbers of infections per DS-TB and MDR-

TB case, respectively, were initialized with estimates from the Mandal study; however, given that these parameters were adjusted by the authors to meet their calibration targets, we performed adjustments to these parameters to meet our calibration targets as well. However, we ensured that the final values of these 'calibrated' parameters remained within the uncertainty intervals specified in the Mandal study.

Treatment success rates, treatment default rates and mortality rates for various DS-TB treatment states under RNTCP were estimated from the 2014 TB India report [2]. The treatment success rate under RNTCP for treatment-naïve MDR-TB patients was estimated from the study by Thomas and colleagues [3], and those for defaulters, non-responders and relapsers were estimated from the study by Joseph and colleagues [4]. The rates at which patients seeking treatment after failure and relapse were estimated from the study by Chandrasekharan and colleagues [5].

Treatment success rates, default rates and mortality rates under non-RNTCP care for both DS-TB and MDR-TB patients were estimated from the literature or calculated using relationships between the corresponding parameters under RNTCP. We describe the calculation of the treatment success rate of defaulted patients seeking re-treatment at non-RNTCP facilities as an example. The treatment success rate for treatment-naïve DS-TB patients seeking treatment at non-RNTCP facilities was estimated as 38% from a study by Uplekar and colleagues [6]. Our search of the literature did not yield any information regarding treatment success rates at non-RNTCP facilities for previously treated patients (defaulters, relapsers or non-responders). Therefore, the treatment success rates for defaulters being treated again at a non-RNTCP facility was estimated by multiplying the success rate for treatment-naïve patients by the ratio of the

treatment success rate for defaulters under RNTCP and the treatment success rate for treatment-naïve patients under RNTCP.

Table S1 below lists the parameters used in the model, their values and source.

Table S1: TB transmission dynamics model parameters

Parameter Name	Symbol	Value	Source
Average number of infections per drug-susceptible (DS) TB case per year	β	13.4	Calibrated/[1]
Average number of infections per MDR-TB case per year	β_{mdr}	2.8	Calibrated/[1]
Per care seeking attempt, probability of seeking care in the public sector (following RNTCP scale-up)	p_{max}	0.30	Calibrated/[1]
Proportion of MDR-TB cases whose drug resistance is recognized at the point of TB diagnosis and who start appropriate treatment	q_{max}	0.05	[1]
Reduction in force of infection owing to previous infection	C	0.5	[1]
Proportion of infections undergoing 'rapid' progression	K	0.15	[1]
Rate of breakdown from remote infection to active disease	h	0.001	[1]
Rate corresponding to the delay from the start of symptoms to the initiation of treatment for new patient (whether in public or private sector) (per year)	r	2.0	[1]
Mean duration of first-line treatment (per year – rate at which patients exit the treatment state)	τ_{FL}	2.0	[1]
Rates of default from RNTCP treatment for treatment-naïve patients, and patients seeking treatment after relapse, recurrence, defaulting and experience previous treatment failure (per year)	$d_{n\text{-}RNTCP}$, $d_{R\text{-}RNTCP}$, $d_{rc\text{-}RNTCP}$, $d_{D\text{-}RNTCP}$, $d_{F\text{-}RNTCP}$	0.06, 0.11, 0.06, 0.17, 0.15	[2] ($d_{n\text{-}RNTCP}$), assumed ($d_{rc\text{-}RNTCP}$), [2] (others)
Rates of default from non-RNTCP treatment for treatment-naïve patients, and patients seeking treatment after relapse, recurrence, defaulting and experience previous treatment failure (per year)	$d_{n\text{-}non}$, $d_{R\text{-}non}$, $d_{rc\text{-}non}$, $d_{D\text{-}non}$, $d_{F\text{-}non}$	0.45 (all)	[6] ($d_{n\text{-}non}$), assumed (others)
Rates of repeat care seeking after default, treatment failure, recurrence, and relapse (per year)	w, f, e, v	4.4, 22, 2 1.80	[1] (w), calibrated (e), [5] (others)
Probability of relapse after treatment success	z	0.1	[7]
Annual recurrence rate	ρ	0.003	[1]
Rate of primary MDR acquisition from new patient treated under RNTCP (per year)	m	0.02	[1]

Rate of primary MDR acquisition from patients treated under R-RNTCP, D-RNTCP, F-RNTCP (per year)	n	0.02	Assumed
Mean duration of second-line treatment (per year - rate at which patients exit the treatment state)	τ_{SL}	0.5	[1]
Spontaneous cure rate (per year)	σ	0.166	[1]
Cure probabilities under RNTCP for treatment-naive DS-TB patients after first-line treatment, and patients seeking treatment after relapse, recurrence, defaulting and experiencing previous treatment failure	α_{pub}, α_{R}, α_{rc}, α_{D}, α_{F}	0.87,0.74, 0.87, 0.66, 0.54	[2] (α_{pub}), assumed (α_{rc}), [2] (others)
Cure probabilities under non-RNTCP for treatment-naive DS-TB patients after first-line treatment, and patients seeking treatment after relapse, recurrence, defaulting and experiencing previous treatment failure	α_{prv}, $\alpha_{R\text{-}non}$, $\alpha_{rc\text{-}non}$, $\alpha_{d\text{-}non}$, $\alpha_{F\text{-}non}$	0.51, 0.43, 0.51, 0.39, 0.32	[6] (α_{prv}), estimated (others)
Cure probabilities under RNTCP for treatment-naive MDR-TB patients after first-line treatment, and patients seeking treatment after relapse, recurrence, defaulting and experience previous treatment failure	α'_{pub}, α'_{R}, α'_{rc}, α'_{D}, α'_{F}	0.38, 0.4, 0.38, 0.30, 0.07	[3] (α'_{pub}), assumed (α'_{rc}), [4] (others)
Cure probability under RNTCP treatment of MDR-TB with second-line treatment	α'_{pub2}	0.48	[2]
Cure probabilities under non-RNTCP for treatment-naive MDR-TB patients after first-line treatment, and patients seeking treatment after relapse, recurrence, defaulting and experience previous treatment failure	α'_{prv}, $\alpha'_{R\text{-}non}$, α'_{rc}, $\alpha'_{D\text{-}non}$, $\alpha'_{F\text{-}non}$	0 (all)	[1] (α'_{prv}), assumed (others)
Rates of default from RNTCP treatment for MDR-TB treatment-naïve patients, and patients seeking treatment after relapse, recurrence, defaulting and experience previous treatment failure (per year)	$d'_{n\text{-}RNTCP}$, $d'_{R\text{-}RNTCP}$, $d'_{rc\text{-}RNTCP}$, $d'_{D\text{-}RNTCP}$, $d'_{F\text{-}RNTCP}$	0.24, 0.33, 0.24, 0.40, 0.43	[3] ($d'_{n\text{-}RNTCP}$), assumed ($d'_{rc\text{-}RNTCP}$), [4] (others)
Rate of default from second-line treatment for MDR-TB under RNTCP (per year)	$d'_{S\text{-}RNTCP}$	0.18	[2]
Rates of default from non-RNTCP treatment for MDR-TB treatment-naïve patients, and patients seeking treatment after relapse, recurrence, defaulting and experience previous treatment failure (per year)	$d'_{n\text{-}non}$, $d'_{R\text{-}non}$, $d'_{rc\text{-}non}$, $d'_{D\text{-}non}$, $d'_{F\text{-}non}$	0.45 (all)	[1] ($d'_{n\text{-}nonRNTCP}$), assumed (others)

Per-capita mortality rate before diagnosis (per year)	μ_{UTB}	0.086 [0.075 - 0.11]	[1]
Mortality rate under RNTCP treatment-naive DS-TB patients after first-line treatment, and patients seeking treatment after relapse, recurrence, defaulting and experience previous treatment failure (per year)	μ_{RNTCP}, μ_{R}, μ_{rc}, μ_{d}, $\mu_{F\text{-}RNTCP}$	0.04, 0.07, 0.04, 0.08, 0.09	[2] (μ_{RNTCP}), assumed (μ_{rc}), [2] (others)
Mortality rate during non-RNTCP treatment-naive DS-TB patients after first-line treatment, and patients seeking treatment after relapse, recurrence, defaulting and experience previous treatment failure (per year)	$\mu_{non\text{-}RNTCP}$, $\mu_{non\text{-}R}$, $\mu_{non\text{-}rc}$, $\mu_{non\text{-}d}$, $\mu_{non\text{-}F}$	0.27 (all)	[1] ($\mu_{non\text{-}RNTCP}$), assumed (others)
Mortality rate during RNTCP for MDR-TB treatment-naive DS-TB patients after first-line treatment, and patients seeking treatment after relapse, recurrence, defaulting and experience previous treatment failure (per year)	μ'_{RNTCP}, μ'_{R}, μ'_{rc}, μ'_{D}, μ'_{F}	0.12, 0.07, 0.12, 0.10, 0.14	[3] (μ'_{RNTCP}), assumed (μ'_{rc}), [4] (others)
Mortality rate during non-RNTCP for MDR-TB treatment-naive DS-TB patients after first-line treatment, and patients seeking treatment after relapse, recurrence, defaulting and experience previous treatment failure (per year)	$\mu'_{non\text{-}RNTCP}$, $\mu'_{non\text{-}R}$, $\mu'_{non\text{-}rc}$, $\mu'_{non\text{-}D}$, $\mu'_{non\text{-}F}$	0.27 (all)	[1] ($\mu'_{non\text{-}RNTCP}$), assumed (others)
Mortality rate during MDR-TB RNTCP treatment under second line treatment (per year)	μ'_{S}	0.22	[2]
Mortality rate following default (per year)	μ_{B}	0.28	[1]
Mortality rate following treatment failure (per year)	μ_{F}	0.28	[1]

References

1. **Mandal S, *et al.*** Counting the lives saved by DOTS in India : a model-based approach. *BMC Medicine* BMC Medicine, 2017; **15**: 1–10.

2. **Central TB Division**. *Revised National Tuberculosis Control Programme, Annual Status Report 2014*. New Delhi, India, 2014.

3. **Thomas, A., Ramachandran, R., Rehaman, F., Jaggarajamma, K., Santha, T., Selvakumar, N., Krishnan, N., Sunder Mohan, N., Sundaram, V., Wares, F. and Narayanan PR**. Management of Multi drug resistance tuberculosis in the field: tuberculosis research centre experience. *Indian Journal of Tuberculosis* 2007; **54**: 117–124.

4. **Joseph P, *et al.*** Influence of drug susceptibility on treatment outcome and susceptibility profile of ‘failures’ to category II regimen. *Indian Journal of Tuberculosis* 2006; **53**: 141–148.

5. **Chandrasekaran, V., Gopi, P.G., Santha, T., Subramani, R. and Narayanan PR**. Status of re-registered patients for tuberculosis treatment under DOTS programme. *Indian Journal of Tuberculosis* 2006; **54**: 11–16.

6. **Uplekar, M., Juvekar, S., Morankar, S., Rangan, S. and Nunn P**. Tuberculosis patients and practitioners in private clinics in India. *International Journal of Tuberculosis and Lung Disease* 1998; **2**: 24–29.

7. **Azhar GS**. DOTS for TB relapse in India: A systematic review. *Lung India* 2012; **29**: 147–153.
